# Unraveling shared and unique dysregulated transcriptomic meta‐programs in synucleinopathies and Alzheimer's disease

**DOI:** 10.1002/alz70855_102704

**Published:** 2025-12-24

**Authors:** Inkyung Jung

**Affiliations:** ^1^ Korea Advanced Institute of Science and Technology, Yuseong‐gu, Daejeon, Korea, Republic of (South)

## Abstract

**Background:**

Synucleinopathies and Alzheimer's disease (AD) share both common and distinct molecular and pathological features, but a comprehensive characterization of these diseases remains elusive.

**Methods:**

We constructed a single‐nucleus and spatial transcriptome atlas of synucleinopathies and AD, analyzing 3.4 million nuclei from 513 post‐mortem human brain samples. Using 167 transcriptomic meta‐programs, we elucidated the complex molecular mechanisms underlying both diseases. We also examined co‐dysregulated meta‐programs across different cell types in each patient.

**Results:**

Notably, we identified 44 disease‐specific meta‐programs, including inflammatory responses in microglia, across synucleinopathies and AD. We further revealed co‐dysregulation of microglia and oligodendrocyte precursor cells (OPCs) in AD. In contrast, synucleinopathies demonstrated co‐dysregulation of astrocytes, microglia, and vascular cells. These co‐dysregulated transcriptomic meta‐programs were verified by spatial transcriptomics using MERFISH.

**Conclusion:**

Our integrative analysis of single‐cell and spatial transcriptomes provides insights into the shared and unique molecular signatures underlying neurodegenerative diseases.

